# Manipulation of the dephasing time by strong coupling between localized and propagating surface plasmon modes

**DOI:** 10.1038/s41467-018-07356-x

**Published:** 2018-11-19

**Authors:** Jinghuan Yang, Quan Sun, Kosei Ueno, Xu Shi, Tomoya Oshikiri, Hiroaki Misawa, Qihuang Gong

**Affiliations:** 1 State Key Laboratory for Mesoscopic Physics and Collaborative Innovation Center of Quantum Matter, Department of Physics, Peking University, Beijing, 100871 China; 2 Research Institute for Electronic Science, Hokkaido University, Sapporo, 001-0021 Japan; 3 Center for Emergent Functional Matter Science, National Chiao Tung University, Hsinchu, 30010 Taiwan; 4 Collaborative Innovation Center of Extreme Optics, Shanxi University, Taiyuan, Shanxi 030006 China

## Abstract

Strong coupling between two resonance modes leads to the formation of new hybrid modes exhibiting disparate characteristics owing to the reversible exchange of information between different uncoupled modes. Here, we realize the strong coupling between the localized surface plasmon resonance and surface plasmon polariton Bloch wave using multilayer nanostructures. An anticrossing behavior with a splitting energy of 144 meV can be observed from the far-field spectra. More importantly, we investigate the near-field properties in both the frequency and time domains using photoemission electron microscopy. In the frequency domain, the near-field spectra visually demonstrate normal-mode splitting and display the extent of coupling. Importantly, the variation of the dephasing time of the hybrid modes against the detuning is observed directly in the time domain. These findings signify the evolution of the dissipation and the exchange of information in plasmonic strong coupling systems and pave the way to manipulate the dephasing time of plasmon modes, which can benefit many applications of plasmonics.

## Abstract (web summary)

Strong coupling between two resonant modes is a prerequisite for many scientifically and technologically interesting effects. Here, Yang et al. demonstrate strong coupling between localized and propagating plasmon modes with a splitting energy of 144 meV and controllable dephasing times.

## Introduction

Localized surface plasmon resonances (LSPRs)—the collective oscillation of charge carriers at the surfaces of metallic nanoparticles—confine light on the nanoscale and can strongly promote the light–matter interactions^1,2^. The most significant properties of LSPRs are their local field enhancement effect and fast damping mechanism, both of which are of great importance in many applications. On the one hand, local field enhancement prompts LSPR-enhanced nonlinear optical processes^3,4^, Purcell enhancement^5–7^, photo harvesting for solar cell applications^8,9^, and artificial photosynthesis^10,11^. On the other hand, the fast damping of LSPRs leads to a short dephasing time of the coherent resonance, which can potentially limit some LSPR-based applications. For example, LSPR-based fluorescence enhancement is analogous to the Purcell enhancement in molecule/cavity systems. The enhancement factor can be described by the Purcell factor, which is proportional to the ratio between the quality factor (*Q*) and the mode volume (*V*). For LSPRs, the dephasing time has a positive correlation to the *Q*. A short dephasing time limits the improvement of the Purcell factor. The applications for sensing also favor larger *Q*^12^, i.e., longer dephasing times. However, the fundamental dipole LSPR of gold (Au) nanoparticles has a dephasing time of only a few femtoseconds^13–15^, and the fast dephasing results from both radiative damping with coupling into far field via scattering process and nonradiative damping that can create electron–hole pairs in metal via either interband or intraband transition^16^. Manipulation and especially prolongation of the dephasing time of LSPR is highly desirable. One way to prolong the dephasing is the excitation of dark or subradiant modes attributed to the reduction of the radiation loss^17,18^. Alternatively, the strong coupling between the plasmon mode and other resonance mode can result in new hybrid modes^9,19–24^, the dephasing time of which can also be manipulated due to exchanging mutual information between the uncoupled modes. Here, we experimentally manipulate the dephasing time of coupled plasmon modes under strong coupling between LSPRs and propagating surface plasmon polaritons (SPPs) directly in the time domain.

Analogous to light–matter interaction between a cavity or plasmon mode and dye molecules or emitters^24–30^, the interaction between LSPRs and SPPs can also be controlled; in the strong coupling regime, the formation of new hybrid LSPR–SPP modes is expected. Several studies have demonstrated the strong coupling between LSPRs and SPPs theoretically and experimentally from the view of far-field spectroscopy^31–34^. However, the far-field spectra carry only limited information about the modification of modes resulting from the strong coupling. The experimental validation in the near field and the direct measurement of the dissipation of the strongly coupled LSPR–SPP modes in the time domain are even more challenging but are indispensable for understanding the essence of strong coupling between LSPRs and SPPs.

In this article, we investigate the strong coupling between the LSPR mode and SPP-Bloch wave, which is a standing wave of propagating SPPs, in multiple domains. An anticrossing behavior observed in the far-field spectra verifies the strong coupling between the LSPR mode and SPP-Bloch wave. More importantly, the near-field spectral properties of the strongly coupled system are investigated by multiphoton photoemission electron microscopy (PEEM), which has been recently proved promising for studying both SPPs and LSPRs^14,35–43^. Here, the near-field spectra, representing the excitation-wavelength-dependent photoemission (PE) intensity curves, visually exhibit normal-mode splitting (similar to Rabi splitting in light–matter strong coupling regimes) and display the extent of coupling visually in the near-field frequency domain. Most importantly, in the time domain, the ultrafast dynamic process can be directly recorded by time-resolved PEEM. In particular, the variation of the dephasing time against the detuning (*E*_SPP_–*E*_LSPR_) reveals the evolution of mode dissipation and exhibits anticrossing behavior. At small detuning, the dephasing time of both LSPR–SPP hybrid modes is demonstrated to be longer than that of the bare LSPR mode. In addition, the near-field enhancement of the hybrid modes is still maintained and is even higher than that of the bare LSPR mode. These findings greatly enhance our understanding of the strong coupling between different plasmon modes and can benefit many plasmonic applications.

## Results

### Structural characterization

**Fig. 1:**
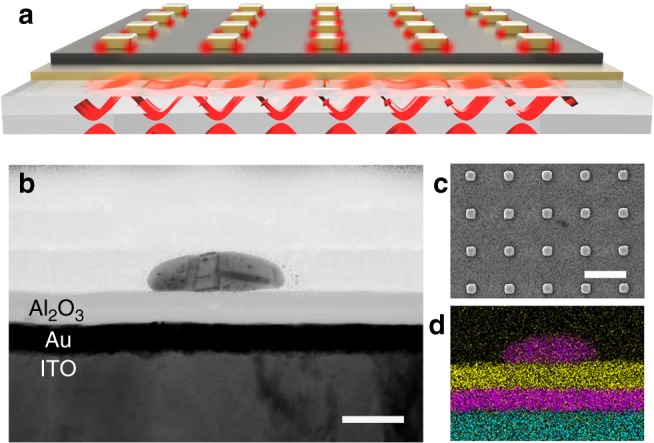
Sample geometry. **a** Schematic diagram for the structure and the mode distribution. The localized surface plasmon resonance (LSPR) mode is located mainly at the interface between the nanoblocks and Al_2_O_3_ layer. The surface plasmon polariton (SPP)-Bloch wave is confined mainly on the lower surface of the Au film. **b** Sectional view of the sample imaged by scanning transmission electron microscope (STEM) (scale bar: 50 nm). **c** Top view of the sample imaged by scanning electron microscope (SEM) (scale bar: 500 nm). **d** Different elements identified using energy-dispersive X-ray spectroscopy (EDS) are presented via specific colors in the sectional view (pink: Au; yellow: Al; blue: In)

The structure designed to realize the strong coupling is shown in Fig. 1. A 20-nm-thick gold film is deposited on an indium-tin-oxide (ITO)-coated glass substrate to support the SPP-Bloch wave. The ITO layer has a thickness of 150 nm, which makes the entire substrate surface suitably conductive for PEEM measurements. Then, a 25-nm-thick Al_2_O_3_ spacer is deposited using the atomic layer deposition technique. The gold square nanoblock arrays are fabricated on the Al_2_O_3_ spacer via electron-beam lithography (EBL), followed by metal sputtering and lift-off, to support the LSPR modes. The sectional view (Fig. 1b) and top view (Fig. 1c) of the sample are acquired by a scanning transmission electron microscope (STEM) and a scanning electron microscope (SEM), respectively. In addition, energy-dispersive X-ray spectroscopy (EDS) is used to mark different elements with a distinct color in the sectional view (Fig. 1d). The nanoblocks of different sizes (side lengths) are designed (100–160 nm) to tune the LSPR energy. Beyond that, the nanoblock array can provide the additional wave vector for the excitation light (**K**_0_) to excite the SPP supported on the thin metal by compensating the momentum mismatch between the excitation light and the SPP modes. The SPP wave vector **K**_SPP_ is satisfied by

1 $${\bf K}_{{\mathrm{SPP}}}{\mathrm{ = }}{\bf K}_0{\mathrm{sin}}\theta _0{{ \pm p}}\frac{{{\mathrm{2\pi }}}}{{D_x}}{\bf u}_x{{ \pm q}}\frac{{{\mathrm{2\pi }}}}{{D_y}}{\bf u}_y$$

where *θ*_0_ is the incident angle, **u**_*x*_ and **u**_*y*_ are the unit reciprocal lattice vectors of a periodic structure, *D*_*x*_ and *D*_*y*_ are the periods, and *p* and *q* are integer numbers determining the SPP propagation direction. Hence, this SPP wave can be interpreted as an SPP-Bloch wave, which is a kind of SPP standing wave sensitive to the period^44,45^. In this way, different periods can be designed (400–600 nm) to tune the energy of the SPP-Bloch wave.

### Experimental far-field spectral property

**Fig. 2:**
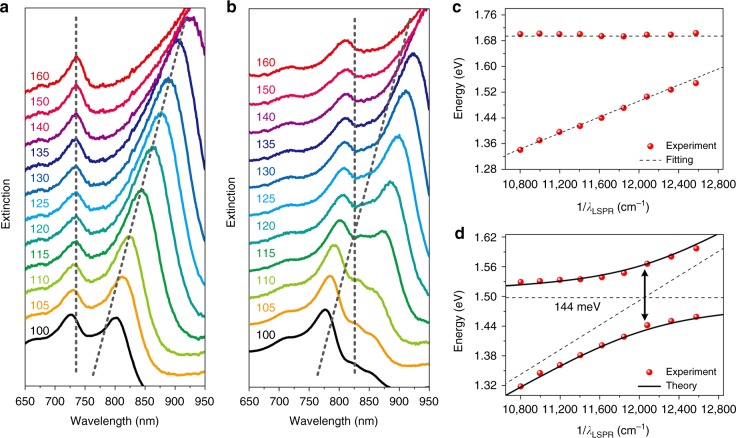
Extinction spectra and energy dispersion. **a**, **b** Extinction spectra of samples with different nanoblock sizes (100–160 nm, indicated by the different color traces) at the fixed period of 400 and 500 nm, respectively. The gray dashed lines indicate the variation trend of the uncoupled localized surface plasmon resonance (LSPR) mode and surface plasmon polariton (SPP)-Bloch wave with increasing nanoblock size. The vertical dashed line represents the SPP-Bloch waves, and the oblique dashed line represents the LSPR modes. **c**, **d** Dispersion of the two branches plotted against the wavenumber for the uncoupled LSPR modes corresponding to **a** and **b**, respectively. The dashed lines correspond to the dispersion of the uncoupled LSPR mode and SPP-Bloch wave. The horizontal line represents the energy of the SPP-Bloch wave, and the oblique line represents the energy of the LSPR mode. The solid lines in **d** correspond to the real part of the eigen-energies of the coupled modes calculated by the coupled oscillator model with a splitting energy of 144 meV at *E*_LSPR_ = *E*_SPP_

The measured extinction spectra of samples with different nanoblock sizes and fixed periods (400 or 500 nm) are presented in Fig. 2a, b, respectively. For the period of 400 nm, the left peak is almost entirely un-shifted, and the right peak undergoes a redshift as the nanoblock size increases. The left and right peaks can be assigned to the SPP Bloch wave and LSPR mode, respectively. Moreover, in this case the two modes cannot couple well with each other, as is clearly shown by the dispersion curves of the two modes (Fig. 2c), where the SPP modes are kept unchanged while the nanoblock sizes change. Similarly, the dissipation of the LSPR mode (*γ*_LSPR_ = 98 meV) and the SPP-Bloch waves (*γ*_SPP_ = 38 meV) can be calculated from the experimental line widths with a period of 400 nm and a nanoblock size of 135 nm. For the period of 500 nm, the dispersion curves (Fig. 2d) extracted from the extinction spectra show an anticrossing behavior and can be fitted by the coupled oscillator model^9,22,26^ (details are shown in the Supplementary Note 1). The splitting energy is calculated as 144 meV at *E*_LSPR_ = *E*_SPP_. Then, we can determine that the interaction potential (*V*) is 78 meV. The results verify that the interaction is in the strong coupling regime because the interaction potential (78 meV) is larger than the average dissipation ($\sqrt {\gamma _{{\mathrm{LSPR}}}^2/2 + \gamma _{{\mathrm{SPP}}}^2/2} = 74\,{\mathrm{meV}}$). The spectra and dispersion curves with other periods are shown in Supplementary Information (Supplementary Figs. 3 and 4). Note that for a thin metal film, the coupling between the SPPs associated with each boundary gives rise to two mixed modes, a symmetric mode and an antisymmetric mode^46,47^. More details about these two modes in our structure are given in Supplementary Note 2. Therefore, the rest peak at the short wavelength range on the extinction spectrum indicates another kind of SPP mode (the symmetric mode). In addition, dual coupling between the LSPR mode and two kinds of SPP-Bloch waves can be observed on the dispersion curves with a period of 550 and 600 nm. Because the coupling efficiency of light scattered into the symmetric mode is much lower than that of light scattered into the antisymmetric mode^47^, the extinction spectrum shows that the peak of the symmetric mode is relatively weak. Hence, in our paper, we focus mainly on the strong interaction between the LSPR mode and the antisymmetric SPP-Bloch wave. It is worth noting that the change of the nanoblock size or the period would also alter the coupling efficiency of the light into the SPP-Bloch wave. However, the impact by the size or period is not significant in this study. Because the change of the size (100–160 nm) and period (400–600 nm) is limited in a small range. Instead, the detuning plays the most important role in the modal strong coupling.

### Simulation results

**Fig. 3:**
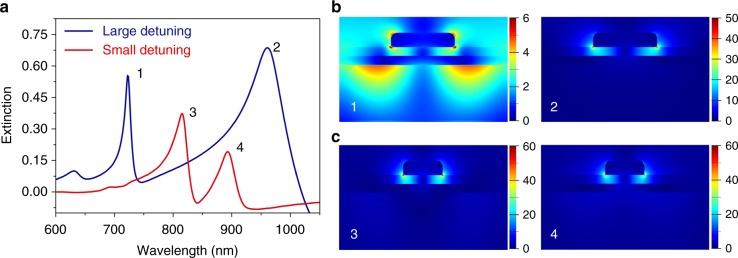
Simulated spectra and electric field distributions. **a** Simulated extinction spectra with large (~396 meV) and small detuning (~19 meV). For the large detuning, the nanoblock size is 150 nm, and the period is 400 nm. For the small detuning, the nanoblock size is 115 nm, and the period is 500 nm. **b**, **c** Electric field (|*E*|) distributions are simulated at 1–4 resonance wavelengths labeled on the extinction spectra

To further understand these modes, we use the finite-difference time domain (FDTD) method to simulate the mode distribution. With the large nanoblock size (150 nm) and the small period (400 nm), two peaks appear on the extinction spectrum (blue line in Fig. 3a). Peak 1 has a narrow line width, and the electric field is confined mainly on the lower surface of the Au film. Peak 2 has a broad line width, and the electric field is located mainly at the interface between the nanoblocks and Al_2_O_3_, with much greater field enhancement, as shown in Fig. 3b. Therefore, we recognize that peaks 1 and 2 represent the SPP-Bloch wave and LSPR mode, respectively, and that the detuning between the LSPR mode and SPP-Bloch wave is large (~396 meV) in this case. Then, when we change the nanoblock size to 115 nm and the period to 500 nm, two main peaks can still be identified on the extinction spectrum (red line in Fig. 3a). However, the two peaks become similar, and similar electric field distributions with high enhancement can be acquired at peaks 3 and 4 (in particular, one order higher than peak 1), as shown in Fig. 3c. Furthermore, we also simulate the field distribution evolution of different peaks in the time domain, as shown in Supplementary Fig. 5. The complete dynamic process is shown in Supplementary Movies 1–4. For peak 1, the SPP-Bloch wave is excited by the incident light scattered by the nanoblock array and then oscillates independently with the decay. Peak 2 refers to the LSPR mode, which is not coupled to the SPP-Bloch wave due to the large detuning. In the case of small detuning of ~19 meV (peaks 3 and 4), the SPP-Bloch wave can be coupled to the LSPR mode; thus, the energy is exchanged reversibly between the two coupled modes. The energy exchange gives rise to a higher near-field enhancement than the single SPP-Bloch wave and a longer oscillation time than the single LSPR mode, which demonstrates that the coupling between the LSPR mode and SPP-Bloch wave modifies the field distribution resulting from the normal-mode splitting with the small detuning. Notably, the near-field enhancement of the two coupled modes (peaks 3 and 4) is both present and, in fact, slightly higher than that of the LSPR mode only (peak 2), which is shown by Fig. 3b, c.

### Experimental near-field spectral property

**Fig. 4:**
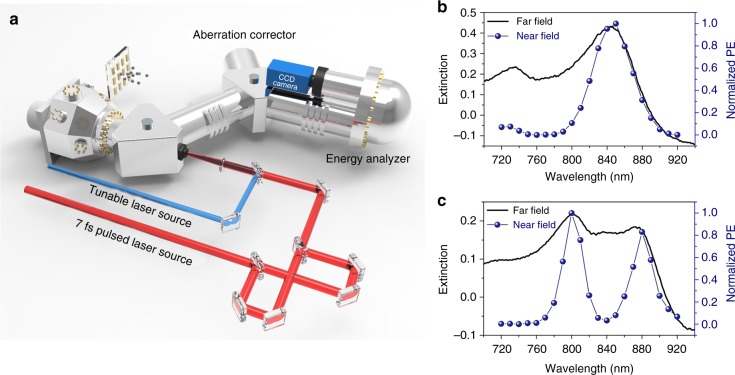
Near-field and far-field measurements. **a** Sketch of the photoemission electron microscopy (PEEM) configuration with two kinds of laser sources. **b**, **c** Far-field spectra (solid lines) and near-field photoemission (PE) intensity curves with different detuning characteristics. In **b**, the 400 nm period and 115 nm nanoblock correspond to the large detuning conditions (221 meV). In **c**, the 500 nm period and 115 nm nanoblock correspond to the small detuning conditions (25 meV)

For far-field spectra, there are always two peaks regardless of the detuning, and it is hard to infer the coupling extent directly. Hence, it is insufficient to illustrate the strong interaction between the LSPR mode and the SPP-Bloch wave using only the far-field spectra. Here, we employ PEEM to investigate the near-field properties of the strong coupling in both frequency and time domain. Previously, PEEM has been reported as a powerful and versatile approach in probing the near field of plasmonic structures from multiple domains. In spatial domain, PEEM images obtained under resonance excitation conditions can be regarded as nonlinear mapping in near field^14,35–43^. In this study, we focus on the frequency and time domain. In our PEEM system (Fig. 4a), two kinds of laser sources are used to measure the near-field PE signal in the frequency domain and time domain, respectively. One is a wavelength tunable (720–920 nm) laser source with a pulse duration of 100 fs. Upon laser illumination onto the sample at normal incidence, the photoemitted electrons can be collected and then integrated to achieve a PE intensity with a specific wavelength. Then, by tuning the wavelength under the condition that the pulse duration and laser power are maintained, we can plot wavelength-dependent PE intensity curves. Because the PE intensity is correlated with the near-field electric field intensity on the sample surface in a nonlinear manner, the wavelength-dependent PE curves can be regarded as near-field spectra to some extent. The other laser source can emit extremely short pulses of 7 fs with a bandwidth above 200 nm. The ultrashort laser pulses were split in a stabilized Mach–Zehnder interferometer into two identical pulses with an adjustable time delay and subsequently focused onto the sample. By using this interferometric pump and probe technique^14,39^, the temporal evolution of PE intensity can be recorded against the delay time between the pump and probe pulses at a frame interval of 0.7 fs, which is chosen as *π*/2 phase delay in carrier wavelength. Thus, the oscillation of the PE signal contains the time information of the modes.

**Fig. 5:**
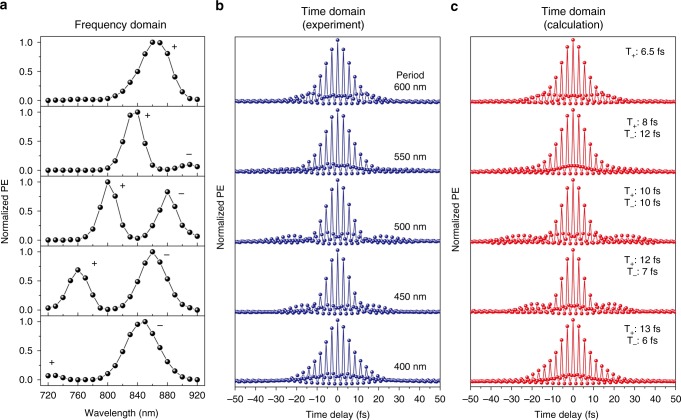
Near-field measurements in both the frequency and time domains. **a** Normalized photoemission (PE) intensity curves against the wavelength in the frequency domain. **b** Normalized PE intensity curves against the time delay in the time domain obtained by time-resolved photoemission electron microscopy (PEEM) measurements. **c** Calculated time-resolved PE signals with the best fitted dephasing time. The nanoblock size is fixed as 115 nm and the period is tuned from 400 to 600 nm

In the frequency domain, the near-field PE intensity curves show different characteristic from the far-field spectra. According to the far-field dispersion curves discussed above, the 400 nm period and 115 nm nanoblock correspond to large detuning (221 meV). As revealed on the PE intensity curve (shown in Fig. 4b), only one main peak can be observed because of the higher near-field enhancement of the LSPR mode at the interface of the Au structures and Al_2_O_3_. More electrons collected by PEEM correspond to a higher peak on the PE intensity curve. In addition, field distributions of the LSPR mode are shown in the PEEM images (Supplementary Fig. 6b). For the SPP-Bloch wave, the electric field located at the lower surface of the Au film cannot efficiently contribute to the PE signal, so the peak of the SPP-Bloch wave is relatively small, and only dim patterns appear in the PEEM image (Supplementary Fig. 6a). When the detuning becomes small (25 meV), as with the sample that has a 500 nm period and 115 nm nanoblock (Fig. 4c), two comparative peaks can be observed on the PE intensity curve. These two peaks represent two coupled modes with comparative near-field enhancement. Correspondingly, similar field distributions can be imaged by PEEM (patterns in Supplementary Figs. 6c and 6d). This result is consistent with the simulated result shown in Fig. 3c. The presence of the two comparative peaks reflects the normal-mode splitting directly. In addition, to show the homogeneous response in our sample, more near-field spectra from the same array are displayed in Supplementary Fig. 7. We compare the near-field spectra from three individual nanoblocks (Supplementary Fig. 7c–e) within the same array and also compare them to that integrated over the whole field of view of 10 μm (Supplementary Fig. 7b). All the four spectral curves give the same spectral peak wavelengths and their shapes are very similar. Considering the nonlinearity of the PE intensity, we can conclude that the spectral response of the structures in the same array is quite homogenous. Moreover, when the energy of the SPP-Bloch wave (Fig. 5a) or LSPR mode (Supplementary Fig. 8a) is tuned, the relative height of the two peaks is also changed. Therefore, the PE intensity curves can visually represent the existence of normal-mode splitting and the extent of the coupling.

### Manipulation of dephasing time

In the strong coupling regime, the energy exchanged reversibly between two coupled modes causes the dissipation to vary. Therefore, the narrow line widths can be measured in the far-field spectra and PE intensity curves in the case of small detuning. The most appropriate way to gain more information about the dissipation of the different modes is to characterize the time-domain response. Here, the ultrafast dynamic process can be measured with the time-resolved PEEM experiment mentioned above (with experimental details as described in our previous paper^14^ and similar to the pioneer works on time-resolved PEEM reported by Kubo et al.^36,37^). As reported before^37^, when the pump and probe pulses overlap, the oscillation of the PE signal is dominated by the interference of the pump and probe pulses at the laser carrier frequency. Above a certain delay time, the pump and probe pulses no longer overlap. The oscillation of the PE signal is dominated by the interference of the excited surface plasmon modes induced by the pump and probe pulses at their individual resonance frequencies. Beyond that, different excited modes modulate the relative intensity and envelope of the PE signal. The above information can be seen in both the experimental time-resolved PE curves (Fig. 5b) and the related movies made from frames using PEEM images at different time delays (an example of a movie recorded for the array with block size of 115 nm and period of 450 nm is provided in Supplementary Movie 5). Furthermore, we can extract the dephasing time of the excited modes by fitting the PE signal.

The excited SP modes can be modeled as damped harmonic oscillators^14,23,48^. Then, the surface plasmon field *E*_*l*_(*t*) can be written as

2 $$E_l(t) \propto {\int}_{ - \infty }^t {\mathop {\sum}\limits_j {\frac{{A_j}}{{\omega _j}}K(t^{\prime})e^{ - \gamma _j(t - t^{\prime})}\sin [\omega _j(t - t^{\prime})]} {\mathrm{d}}t^{\prime}}$$

where *A*_*j*_ is the oscillator strength, *γ*_*j*_ = 1/2*τ*_*j*_ is the damping of the resonance *ω*_*j*_ (*τ*_*j*_ denotes the dephasing time), and *K*(*t*) = *E*_0_(*t*) + *E*_0_(*t* + *t*_d_) denotes the driving force. Here, *t*_d_ is the time delay between the pump and probe pulses and $E_0(t) = {\mathrm{sech}}\left[ {2\ln \left( {1 + \sqrt 2 } \right)t/{T}_0} \right]\cos (\omega _0t)$, *T*_0_ (7 fs) and *ω*_0_ (820 nm) denote the pulse duration and central frequency of the laser pulse. The oscillator strength (*A*_*j*_) and resonance frequency (*ω*_*j*_) can be defined by the PE intensity curves. Because the PE is a multiphoton process, the PE intensity can be given as

3 $$I(t_{\mathrm{d}}) \propto {\int}_{ - \infty }^{ + \infty } {\left| {E_l(t)} \right|^{2N}{\mathrm{d}}t}$$

where *N* denotes the nonlinear order of the PE validated by the equation $N = \log _4\left( {2I(0)/I(\infty )} \right) \approx 3$^14^. It should be noted that this nonlinear order *N* is lower than the previous value we obtained for Au nanoblocks. A brief discussion about the nonlinear order is also given in Supplementary Note 3. In the calculations, the time interval is also chosen as 0.7 fs to allow for the point-by-point comparison with the experimental data. By comparing the calculated (Fig. 5c) and experimental (Fig. 5b) curves, we can obtain the dephasing time of different modes. Based on the PE intensity curves in the frequency domain (Fig. 5a), the detuning is tuned from large to small and then to large again as the period is changed. For the small period (400 nm) corresponding to the large detuning (221 meV), the right peak is more likely the LSPR mode because of the wider line width. Similarly, in the time domain, the LSPR-like mode has a much shorter dephasing time (6 fs) than the SPP-like mode (13 fs). It is also noted that the dephasing time of the SPP-like mode is only 13 fs, which is similar to that reported for Au films with periodic nanohole arrays^49^, but still substantially shorter than that of the usual SPP mode supported on flat Au films where nonradiative losses dominate^50^. We attribute the dephasing time of approximately 13 fs to the nonradiative losses and the extra losses due to the coupling out of some SPP energy by the Au nanoblock array. The short dephasing time means that the electrons cannot maintain coherent oscillation for an extended time. Therefore, the shorter dephasing time corresponds to the larger dissipation and higher proportion of the LSPR mode. Increasing the period, the two comparative peaks signify strong coupling, which would allow energy exchange between the two coupled modes and reduce the average dissipation. Accordingly, the average longer dephasing time in the time-resolved PEEM experiment directly confirms that this is the case. In particular, for the small detuning of 25 meV (period of 500 nm), an equal dephasing time of 10 fs is measured. For the larger period (600 nm), the detuning becomes large again (−92 meV) with a redshift of the SPP-Bloch wave. Here, the left peak is more similar to the LSPR mode and has the shorter dephasing time (6.5 fs). Therefore, the near-field results in the frequency and time domain measured by PEEM corroborate each other.

## Discussion

**Fig. 6:**
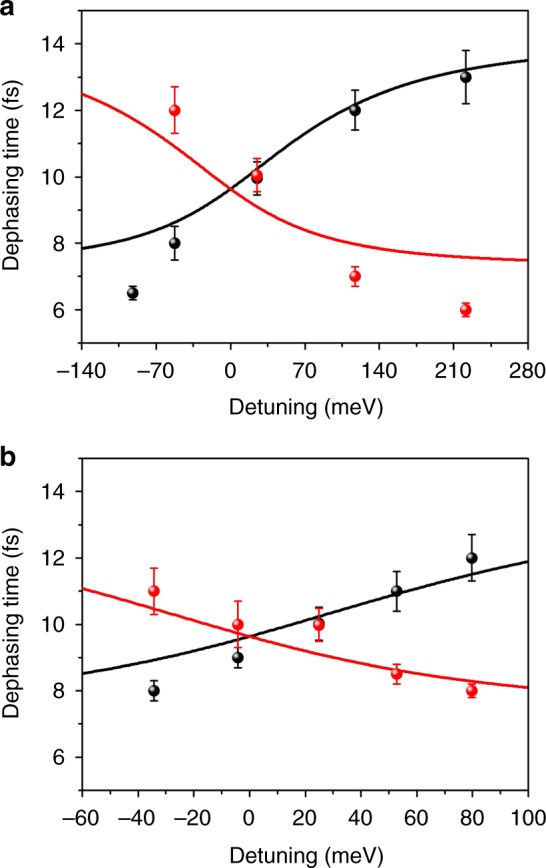
Evolution of the dephasing time against the detuning. **a** Solid lines present the dephasing time extracted from the far-field line width in Supplementary Fig. 9a. The scattered dots are the dephasing time measured by photoemission electron microscopy (PEEM) corresponding to results in Fig. 5. **b** Solid lines present the dephasing time extracted from the far-field line width in Supplementary Fig. 9b. The scattered dots are the dephasing time measured by PEEM corresponding to the results shown in Supplementary Fig. 8. The red and black colors are used to distinguish the different branches. The error bars are the s.d. estimated by the maximum difference of several fitted results

Moreover, we compare the far-field and near-field results to confirm the reliability of our PEEM measurements. The expected far-field line widths (Supplementary Fig. 9) of the coupled modes can be calculated from the imaginary part of the eigen-energies according to the coupled oscillator model. For each branch, as the detuning changes, the line width evolves from large to small and from small to large, which signifies the evolution of the proportion of the LSPR mode and the SPP-Bloch wave in the coupled modes from the perspective of the far-field spectra. According to the relation $\Delta \omega _j \cdot \tau _j = 1$^13^, the dephasing time can also be extracted from the far-field line width (the solid lines in Fig. 6). When the experimental dephasing times (the dots in Fig. 6a, b) acquired from the near-field results in Fig. 5 and Supplementary Fig. 8 are compared, the same variation trend of the far-field and near-field results demonstrates the validity of our approach.

In conclusion, we have demonstrated the strong coupling between the LSPR mode and SPP-Bloch wave based on the far-field spectra with the normal-mode splitting of 144 meV. Furthermore, the near-field properties of the coupled systems are investigated by PEEM in both the frequency and time domains. In the frequency domain, the relative height of the peaks on the wavelength-dependent near-field PE intensity curves visually shows the extent of the coupling. In the time domain, the dynamic process, which contains the dephasing time of different coupled modes, is recorded by the oscillation of the PE signal against the time delay. Importantly, the evolution of the proportion of the LSPR mode and SPP-Bloch wave in the coupled mode can be inferred from the variation of the dephasing time. We suggest that the investigation of the near-field and dynamical properties in this work is applicable to various strong coupling systems and supplements the research of strong coupling from the viewpoint of near field in both the frequency and time domains. The results also provide direction for manipulating the dephasing time of surface plasmons within the plasmonic frame.

## Methods

### Sample fabrication and characterization

A thin Au film was deposited on glass substrates coated with an approximately 150-nm-thick ITO layer via sputtering (MPS-4000, ULVAC). The Al_2_O_3_ layer was deposited by atomic layer deposition (Sunnale-R150, Picosun) at 200 °C for 250 cycles. The nanoblock array was fabricated by using EBL and metal evaporation techniques. The substrate was then sequentially cleaned with acetone, methanol, and ultrapure water in an ultrasonic bath (with each step lasting 5 min). A conventional copolymer resist (ZEP520A, Zeon Chemicals) diluted with a ZEP thinner (1:1) was then spin-coated onto the substrate at 1000 rpm for 10 s and at 4000 rpm for 90 s. The substrate was subsequently prebaked on a hot plate for 2 min at 150 °C. In this study, a high-resolution EBL system (ELS-F125-U, Elionix) operating at 125 kV was used for sample fabrication; the EBL was conducted at a current of 50 pA. After the development, a 2-nm-thick titanium layer was first deposited via sputtering (MPS-4000, ULVAC) as the adhesive layer, followed by the deposition of a 40-nm-thick Au film. Lift-off was performed by successively immersing the sample in anisole, acetone, methanol, and ultrapure water in an ultrasonic bath. Morphologies were analyzed using field-emission scanning electron microscopy (JSM-6700FT, JEOL). Far-field spectral properties were analyzed using a spectrophotometer (PMA-11, Hamamatsu) coupled with a transmission microscope (BX51, Olympus).

### Numerical simulation

We used a FDTD solutions software package (Lumerical, Inc.) to simulate the far-field spectra and the electric field distribution in the near field. The optical properties of Au were obtained using data from Johnson and Christy^51^. The ITO-coated glass substrate and Al_2_O_3_ were assumed to behave as a dielectric material with an average refractive index of *n* = 1.6. The plane wave light source was injected onto Au structures from the air side. In the light propagation direction, the perfectly matched layer boundary conditions were imposed. In the plane perpendicular to the light propagation direction, periodic boundary conditions were applied on each boundary and the simulation region was chosen to be the same as one unit of the array. The mesh size was chosen as 1 nm after convergence tests. A power and profile monitor located in the substrate was used to calculate the transmission. Another power and profile monitor was placed along the light propagation direction to record the cross-section of the field distribution.

## Electronic supplementary material

Supplementary Information

Description of Additional Supplementary Files

Supplementary Movie 1

Supplementary Movie 2

Supplementary Movie 3

Supplementary Movie 4

Supplementary Movie 5

## Data Availability

The authors declare that the data supporting the findings of this study are available from the corresponding authors upon reasonable request.
