# Supplementary material for: Manipulation of the dephasing time by strong coupling between localized and propagating surface plasmon modes
Source: Nat Commun. 2018 Nov 19;9:4858. doi: 10.1038/s41467-018-07356-x (PMC6242842; doi:10.1038/s41467-018-07356-x)
Supplement: Supplementary file 1 — Supplementary Information [file 41467_2018_7356_MOESM1_ESM.pdf]

Supplementary Information for

Manipulation of the dephasing time by strong coupling between localized and propagating surface plasmon modes

Yang *et al.*

Supplementary Note 1: Coupled oscillator model

The eigen-energies of coupled modes can be calculated by the coupled oscillator model:

$$\begin{pmatrix} E_{\mathrm{LSPR}} - \mathrm{i}\hbar\Gamma_{\mathrm{LSPR}} & V \\ V & E_{\mathrm{SPP}} - \mathrm{i}\hbar\Gamma_{\mathrm{SPP}} \end{pmatrix} \begin{pmatrix} \alpha \\ \beta \end{pmatrix} = E \begin{pmatrix} \alpha \\ \beta \end{pmatrix} \tag{1}$$

Here, E_{LSPR} (E_{SPP}) is the energy of the LSPR mode (SPP-Bloch wave), $\hbar\Gamma_{\mathrm{LSPR}}$ ($\hbar\Gamma_{\mathrm{SPP}}$) is the dissipation extracted from the experimental line width, V is the interaction potential and α , β construct the eigenvector. Then the eigen-energies can be written

$$E_{\pm} = \frac{E_{\mathrm{LSPR}} + E_{\mathrm{SPP}}}{2} - \frac{\mathrm{i}(\hbar\Gamma_{\mathrm{LSPR}} + \hbar\Gamma_{\mathrm{SPP}})}{2} \pm \frac{\sqrt{(E_{\mathrm{LSPR}} - E_{\mathrm{SPP}} - \mathrm{i}\hbar\Gamma_{\mathrm{LSPR}} + \mathrm{i}\hbar\Gamma_{\mathrm{SPP}})^2 + 4V^2}}{2} \tag{2}$$

and the Rabi splitting energy at $E_{\mathrm{LSPR}} = E_{\mathrm{SPP}}$ can be calculated as $\sqrt{4V^2 - (\hbar\Gamma_{\mathrm{LSPR}} - \hbar\Gamma_{\mathrm{SPP}})^2}$.

Supplementary Note 2: Symmetric mode and antisymmetric mode

For the thick Au film, both interfaces on the top and bottom can support the SPP mode independently. When the dielectric materials on the top and bottom have identical dielectric properties, these two SPP modes are degenerate. But when the Au film becomes thin, the SPP modes on the top and bottom interfaces would interact with each other to form the mixed modes. As reported in two references[1-3], where it was further explained that there are two kinds of modes guided by the thin film, one symmetric and one antisymmetric with respect to their field distributions, whether the dielectric materials on the top and bottom are the same or not.

In our structure, because of the 20-nm-thick Au film, the SPP modes residing on either side of the Au film cannot exist independently and would interact with each other to form the mixed modes (symmetric mode and antisymmetric mode). The FDTD simulated electric field (|E|) distributions of two modes are shown in Supplementary Figs. 1a and 1b with the block size of 150 nm and the period of 400 nm. Asymmetric structure makes the electric field mainly locate on the bottom side of the Au film. Moreover, the coupling efficiency of light scattered into the symmetric mode is much lower than that of light scattered into the antisymmetric mode[4]. Hence, the antisymmetric mode has the stronger field distribution and larger affected area. To distinguish these two modes (antisymmetric and symmetric), the simulated magnetic field $|H_y|$ distributions are presented in Supplementary Figs. 1c and 1e, and $|H_y|$ mode profiles along the dash lines in Supplementary Figs. 1c and 1e are plotted in Supplementary Figs. 1d and 1f. According to the references[1, 3], for the symmetric mode the transverse field does not exhibit a zero inside the metal

film - conversely the antisymmetric mode has a zero in its transverse field inside the film. The $|H_y|$ mode profile in Supplementary Fig. 1d (1f) with (without) a zero inside the Au film clearly shows the antisymmetric (symmetric) mode distribution.

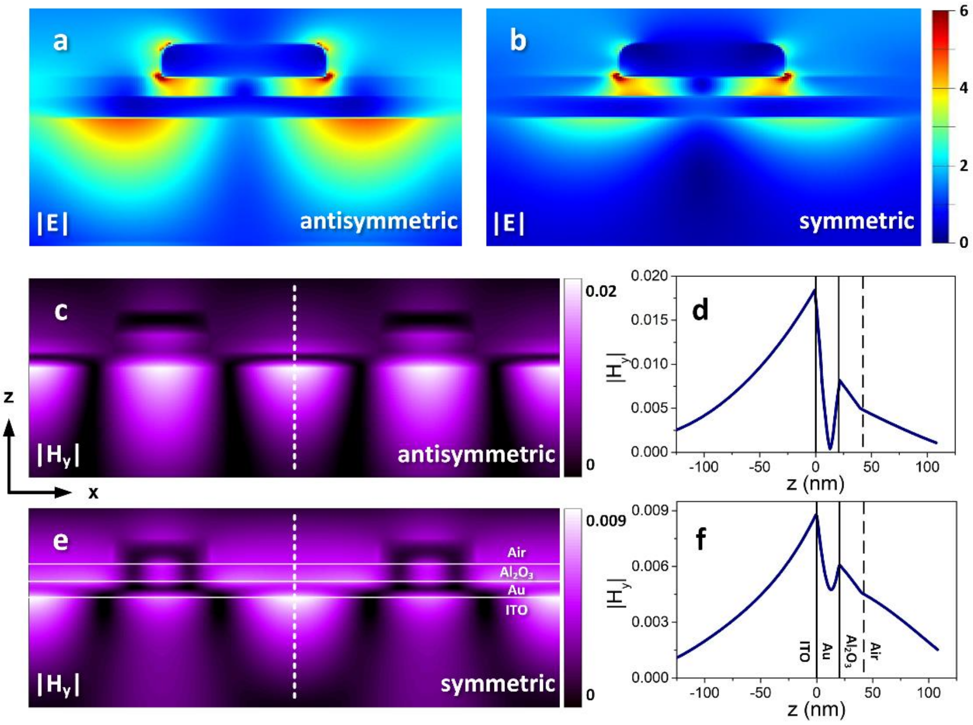

Supplementary Figure 1 | The simulated electric and magnetic field distributions a and b, Electric field (|E|) distributions of the antisymmetric and symmetric modes with the block size of 150 nm and the period of 400 nm. **c and e,** The corresponding magnetic field ($|H_y|$) distributions of the antisymmetric and symmetric mode. **d and f,** $|H_y|$ mode profiles along the dash lines in **c** and **e**.

Supplementary Note 3: Nonlinear photoemission order

The determination of the nonlinear photoemission order (N) as 3 was based on the experimental interferometric time-resolved photoemission (PE) intensity curves using the formula ($N = \log_4(\frac{2I_{\mathrm{PE}}(0)}{I_{\mathrm{PE}}(\infty)})$), which derives from the following process. When the time delay is 0, two pulses would have completely constructive interference. In this case, the photoemission intensity (I_{PE}) can be obtained by $I_{\mathrm{PE}}(0) \propto (I)^N$, where I represents the excitation laser intensity before the Mach-Zehnder interferometer (Supplementary Fig. 2a). When the time delay becomes long enough, there is no interference either between both the pump and probe optical fields or between the two plasmon fields, because when the probe beam arrives, the plasmon field of the pump beam has been totally dephased out. Thus, the photoemission intensity can be thought to result from the incoherent sum of the photoemission from pump and probe beams independently, and in this case both pump and probe light intensity before PEEM should be $I/4$ because of the beam splitting mirror (Supplementary Fig. 2b). Therefore, the photoemission intensity can be expressed as $I_{\mathrm{PE}}(\infty) \propto 2 \times (I/4)^N$. According to these two equations, we can get the nonlinear photoemission order $N = \log_4(\frac{2I_{\mathrm{PE}}(0)}{I_{\mathrm{PE}}(\infty)})$. A typical interferometric time-resolved PE curve is shown in Supplementary Fig. 2c. In this work, the $I(\infty)$ was chosen as the PE intensity at the long time delay of 55 fs, $I_{\mathrm{PE}}(\infty) \approx I_{\mathrm{PE}}(55\ \mathrm{fs}) = 0.032$. Therefore, the $N = \log_4\left(\frac{2I_{\mathrm{PE}}(0)}{I_{\mathrm{PE}}(\infty)}\right) = \log_4\left(\frac{2}{0.032}\right) \approx 2.983$.

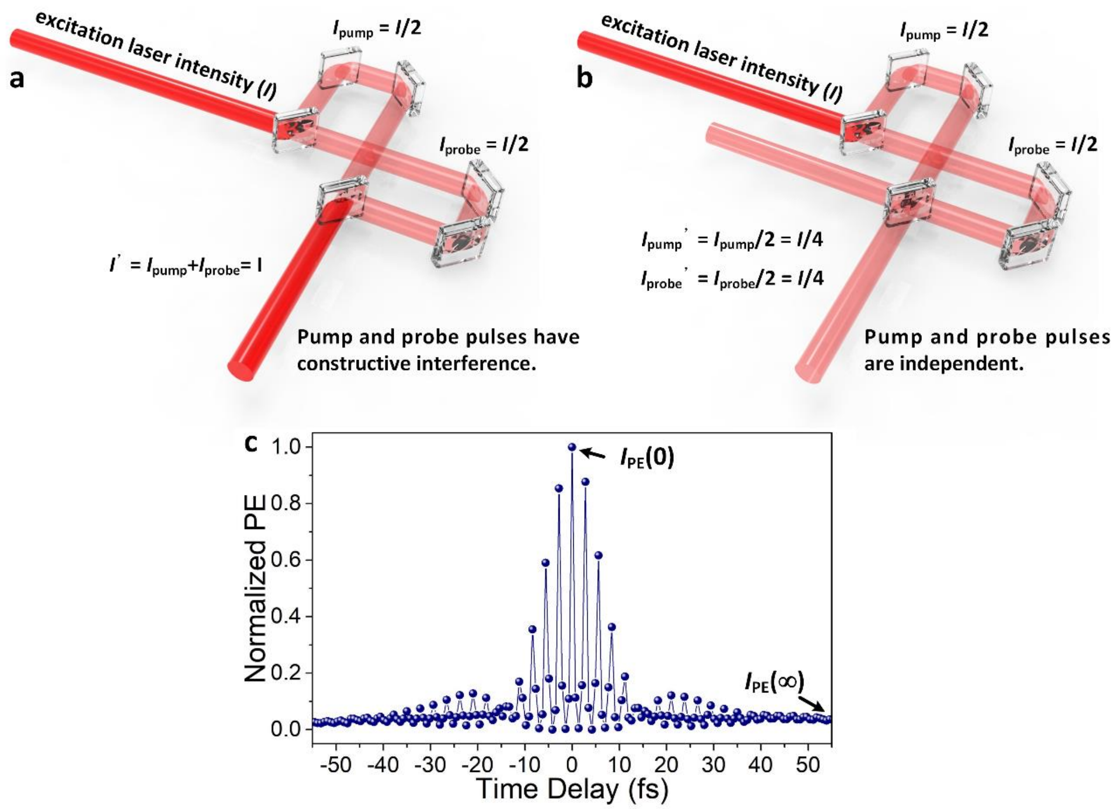

Supplementary Figure 2 | The light path in Mach-Zehnder interferometer and the time-resolved photoemission curve a, The light path with the time delay of 0. **b,** The light path with the time delay of infinity. **c,** The interferometric time-resolved photoemission (PE) curve for structures with the block size of 115 nm and the period of 500 nm. I_{pump} and I_{probe} represent the laser intensity inside the Mach-Zehnder (MZI) interferometer (after the first beam splitter) for the pump beam and the probe beam, respectively. I', I_{pump}', and I_{probe}' represent the corresponding laser intensity after the MZI interferometer.

It should be noted that the nonlinear order N often displays the variation between 3 and 5 for Au nanostructures. For examples, it was ever reported to be 3[5], 3.2[6], ~ 3.7[7, 8]. Very recently it was even reported to reach a high value of 4.5[9]. Some variations in the nonlinear order for different experiments may result from different laser conditions, different plasmon resonance wavelengths,

different near-field intensity, as well as local variations of the gold work function due to substrate effects and crystalline facets. We consider the lower *N* obtained in this study than that in our previous work might be due to the different materials that locate below the Au nanostructures. In our previous study[8], the substrate is ITO-coated glass and thus the material below Au nanostructures is ITO. However, in this study, due to the multiple layer configuration, the base material for Au nanostructures is Al_2O_3 deposited by the ALD technique. The difference in *N* may result from the difference in the effective work function of the sample surface due to different substrates. In addition, the different near-field intensity would also affect the nonlinearity.

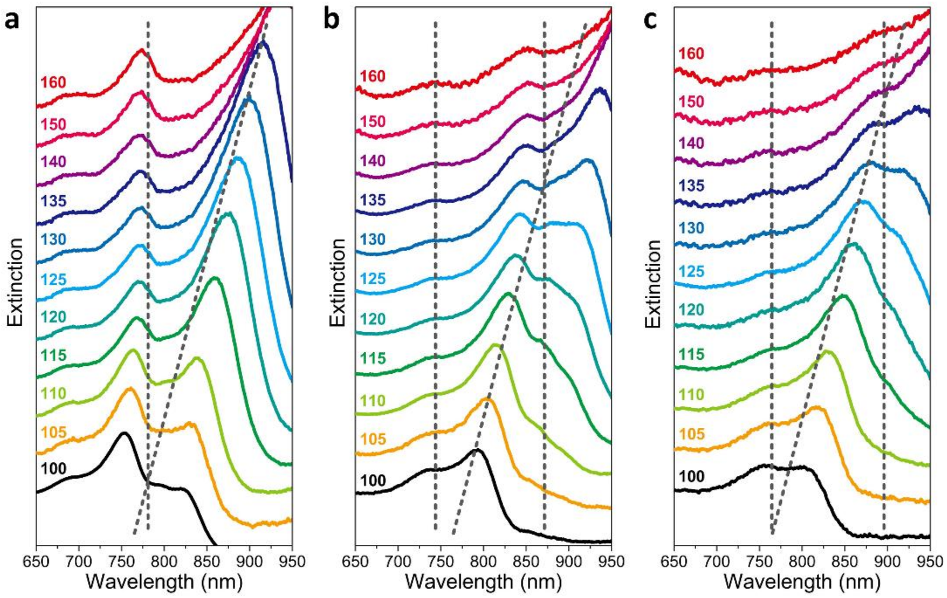

Supplementary Figure 3 | Extinction spectra with different nanoblock sizes and periods a, Extinction spectra with different nanoblock sizes (100 nm to 160 nm, indicated by the different color traces) at the fixed period of 450 nm. **b, c,** Periods are fixed as 550 nm and 600 nm. The gray dashed lines indicate the variation trend of uncoupled localized surface plasmon resonance (LSPR) mode and surface plasmon polariton (SPP)-Bloch wave with the increasing nanoblock size. The vertical dashed lines represent the two kinds of SPP-Bloch waves and the oblique dashed lines represent the LSPR mode.

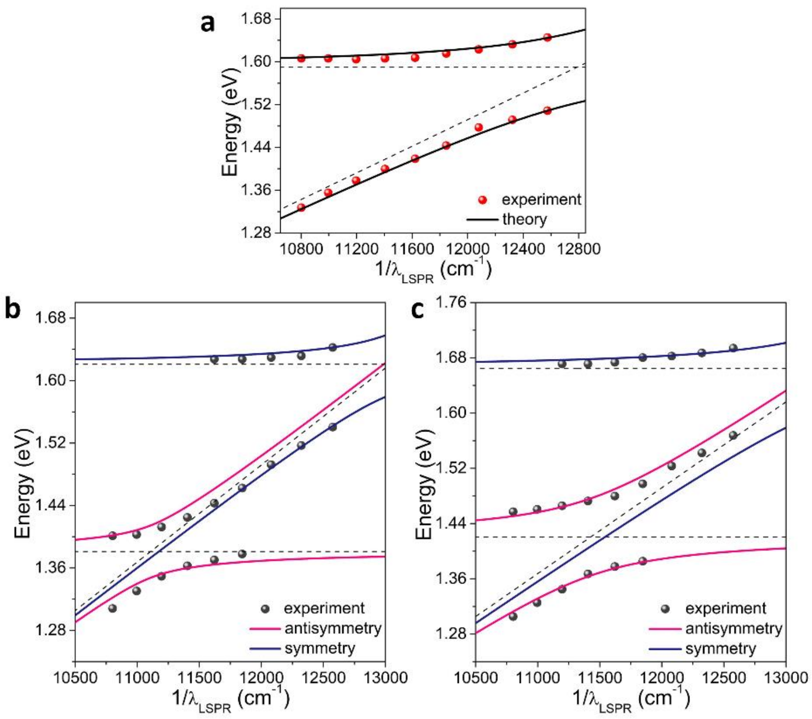

Supplementary Figure 4 | The energy dispersion against the wavenumber a, b, c, Dispersion with different periods 450 nm, 550 nm and 600 nm. The horizontal dashed lines represent the dispersion of uncoupled surface plasmon polariton (SPP)-Bloch waves and the oblique ones represent the dispersion of uncoupled localized surface plasmon resonance (LSPR) mode. The solid lines correspond to the real part of the eigen-energies of coupled modes calculated by the coupled oscillator model.

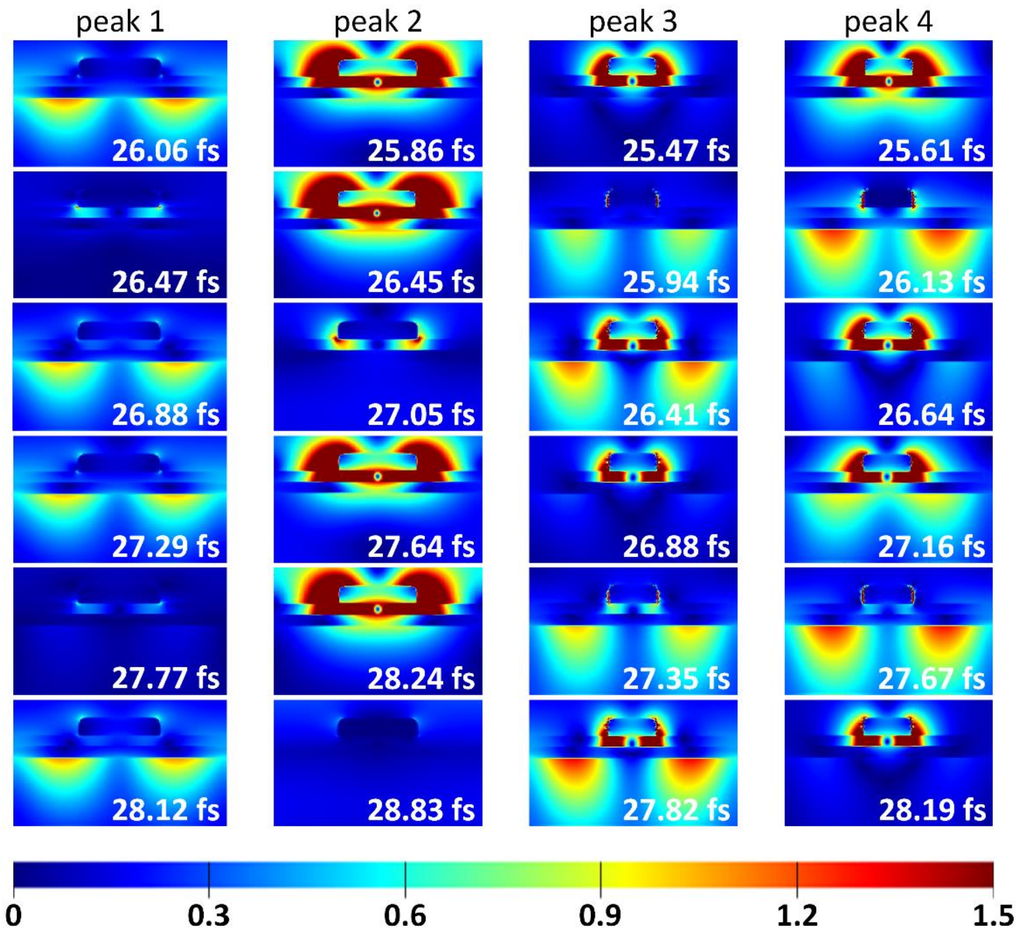

Supplementary Figure 5 | Distribution evolution of different peaks in time domain "Peak 1" represents the surface plasmon polariton Bloch (SPP-Bloch) wave with the block size of 150 nm and the period of 400 nm; "Peak 2" represents the localized surface plasmon resonance (LSPR) mode with the block size of 150 nm and the period of 400 nm; "Peak 3" represents one coupled mode with the block size of 115 nm and the period of 500 nm. "Peak 4" represents another coupled mode with the block size of 115 nm and the period of 500nm. For peaks 1 and 2, the modes oscillate independently because of the large detuning. While in the case of small detuning (peaks 3 and 4), the energy would be exchanged reversibly between two coupled modes.

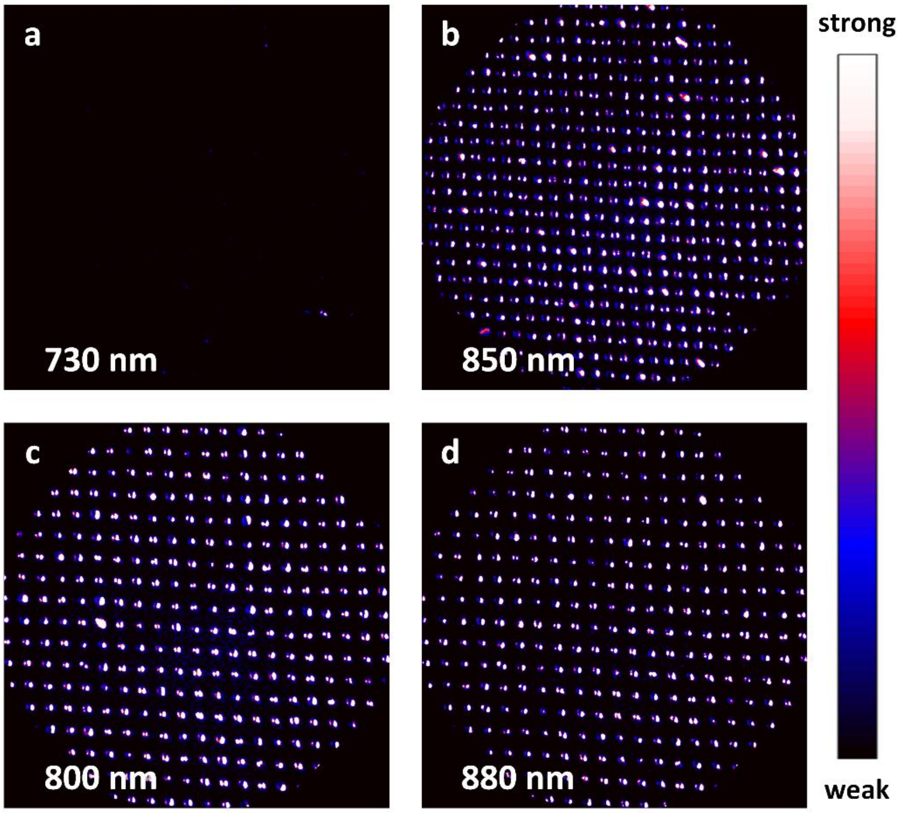

Supplementary Figure 6 | Normalized images with different excitation wavelengths and structures a, b, For the 115 nm nanoblocks with the period of 400 nm, images are measured at the excitation wavelength of 730 nm (**a**) and 850 nm (**b**) by the photoemission electron microscopy (PEEM). **c, d,** For the 115 nm nanoblocks with the period of 500 nm, PEEM images are measured at the excitation wavelength of 800 nm (**c**) and 880 nm (**d**).

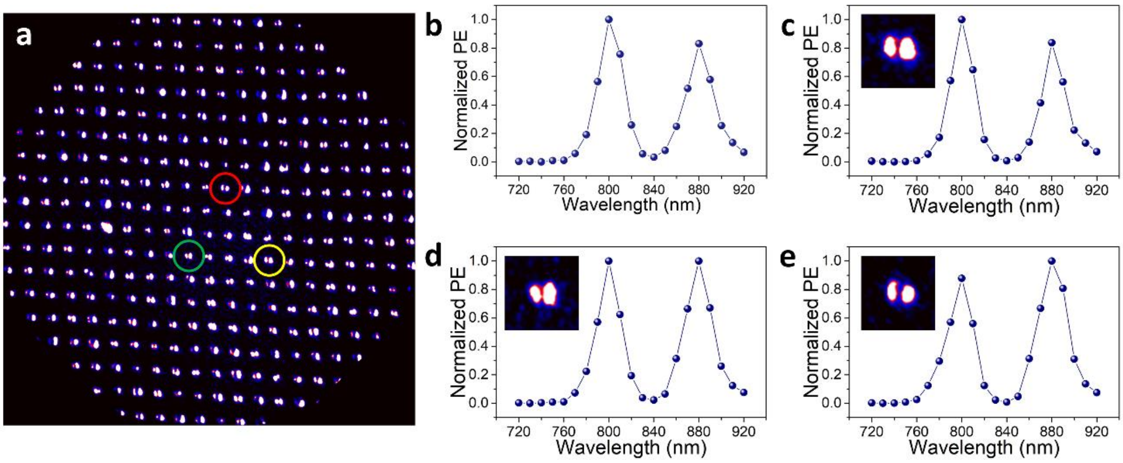

Supplementary Figure 7 | Near-field mapping and near-field spectra a, Near-field mapping of localized surface plasmon resonance (LSPR) with a field of view (FOV) of 10 μm (Block size: 115 nm; Period: 500 nm; Wavelength: 800 nm). **b,** Near-field spectrum integrated over the whole FOV of 10 μm. **c,** Near-field spectrum of the individual nanoblock in a yellow circle. **d,** Near-field spectrum of the individual nanoblock in a green circle. **e,** Near-field spectrum of the individual nanoblock in a red circle.

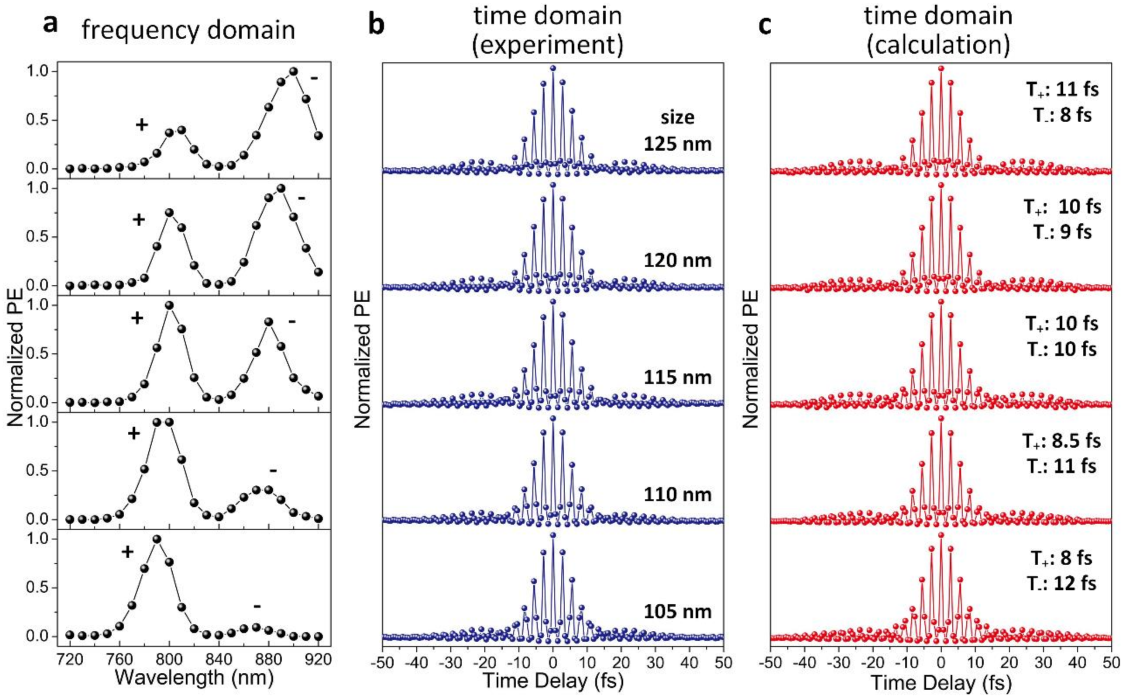

Supplementary Figure 8 | Near-field measurements in both the frequency and time domain a, Normalized photoemission (PE) intensity curves against the wavelength in the frequency domain. **b,** Normalized PE intensity curves against the time delay in the time domain obtained by time-resolved photoemission electron microscopy measurements. **c,** Calculated time-resolved PE signals with the best fitted dephasing time. (The period is fixed as 500 nm and the nanoblock size is tuned from 105 nm to 125 nm).

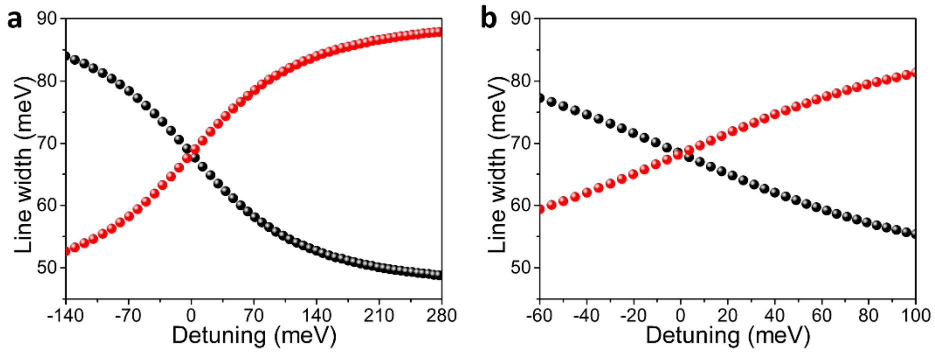

Supplementary Figure 9 | Evolution of the line width calculated by the coupled oscillator model a, The detuning variation corresponds to the change of the structure period. **b,** The detuning variation corresponds to the change of the nanoblock size.

Supplementary References

1. Burke, J. J., Stegeman, G. I. & Tamir, T. Surface-polariton-like waves guided by thin, lossy metal-films. *Phys. Rev. B* **33,** 5186-5201 (1986).

2. Zervas, M. N. Surface-plasmon polariton waves guided by thin metal films. *Opt. Lett.* **16,** 720-722 (1991).

3. Sarid, D. Long-Range Surface-Plasma Waves on Very Thin Metal Films. *Phys. Rev. Lett.* **47,** 1927-1930 (1981).

4. Sondergaard, T., Siahpoush, V. & Jung, J. Coupling light into and out from the surface plasmon polaritons of a nanometer-thin metal film with a metal nanostrip. *Phys. Rev. B* **86,** 085455 (2012).

5. Douillard, L., *et al.* Short Range Plasmon Resonators Probed by Photoemission Electron Microscopy. *Nano Lett.* **8,** 935-940 (2008).

6. Grubisic, A., Schweikhard, V., Baker, T. A. & Nesbitt, D. J. Coherent Multiphoton Photoelectron Emission from Single Au Nanorods: The Critical Role of Plasmonic Electric Near-Field Enhancement. *ACS Nano* **7,** 87-99 (2013).

7. Sun, Q., *et al.* Direct imaging of the near field and dynamics of surface plasmon resonance on gold nanostructures using photoemission electron microscopy. *Light-Sci Appl.* **2,** e118 (2013).

8. Sun, Q., *et al.* Dissecting the Few-Femtosecond Dephasing Time of Dipole and Quadrupole Modes in Gold Nanoparticles Using Polarized Photoemission Electron Microscopy. *ACS Nano* **10,** 3835-3842 (2016).

9. Sivis, M., *et al.* Continuous-wave multiphoton photoemission from plasmonic nanostars. *Commun. Phys.* **1,** 13 (2018).
