# Supplementary material for: Manipulation of the dephasing time by strong coupling between localized and propagating surface plasmon modes
Source: Nat Commun. 2018 Nov 19;9:4858. doi: 10.1038/s41467-018-07356-x (PMC6242842; doi:10.1038/s41467-018-07356-x)
Supplement: Supplementary file 2 — Description of Additional Supplementary Files [file 41467_2018_7356_MOESM2_ESM.pdf]

Supplementary Movie 1 | The dynamic process of peak 1. The field distribution evolution is simulated at the wavelength of the peak 1. The surface plasmon polariton (SPP)-Bloch wave is excited by the incident light scattered by the nanoblock array and then oscillate independently with the decay.

Supplementary Movie 2 | The dynamic process of peak 2. The field distribution evolution is simulated at the wavelength of the peak 2. The localized surface plasmon resonance (LSPR) mode is excited directly by the incident light but cannot induce the surface plasmon polariton (SPP)-Bloch wave owing to the large detuning. Then the LSPR mode would oscillate independently with the decay.

Supplementary Movie 3 | The dynamic process of peak 3. The field distribution evolution is simulated at the wavelength of the peak 3. Because of the small detuning, the surface plasmon polariton (SPP)-Bloch wave and localized surface plasmon resonance (LSPR) mode can be both excited and the energy would be exchanged reversibly between two coupled modes.

Supplementary Movie 4 | The dynamic process of peak 4. The field distribution evolution is simulated at the wavelength of the peak 4. This evolution is analogous with that in the Supplementary movie 3 owing to the strong coupling.

Supplementary Movie 5 | The evolution of the photoemission intensity against the time delay. The block size is 115 nm and period is 450 nm. The left side of the movie shows the plot of the photoemission (PE) intensity as the function of the delay time. The right side of the movie shows the evolution of the PE distribution with the delay time. Each frame corresponds to one point in the plot shown in the left side. The oscillation of the PE signal can be observed due to the interference between the pump and probe pulses or the excited plasmon fields.
